# Key genes affecting the progression of nasopharyngeal carcinoma identified by RNA-sequencing and bioinformatic analysis

**DOI:** 10.18632/aging.203521

**Published:** 2021-09-20

**Authors:** Yihong Wang, Manyi Li, Yan Guo, Haiping Huang, Xuelin Dong, Yangguang Sun, Jisheng Liu

**Affiliations:** 1 Department of Otolaryngology, The First Affiliated Hospital of Soochow University, Soochow 215000, Jiangsu Province, China

**Keywords:** nasopharyngeal carcinoma, differentially expressed genes, protein encoding genes, protein-protein interaction network, progression

## Abstract

Background: The present work was conducted to screen the potential biomarkers affecting nasopharyngeal carcinoma (NPC) progression through RNA-sequencing (RNA-seq), bioinformatic analysis and functional experiments.

Materials and Methods: Six normal samples and five NPC clinical samples were collected for RNA-seq analysis. The expression levels in both groups were determined through student’s *t*-test. We identified genes of *P* < 0.01 as the differentially expressed genes (DEGs). In addition, gene set enrichment analysis (GSEA) was conducted. Afterwards, STRING V10 database was employed to extract protein interactions among the DEGs. Later, we established a protein-protein interaction (PPI) network, and used the Cytoscape software for network visualization. qRT-PCR was conducted to verify hub genes from clinical samples. Then, the function of CXCL10 in cell proliferation, apoptosis, invasion and migration was evaluated.

Results: A total of 2024 DEGs were identified, among which, 1449 were down-regulated and 575 were up-regulated. The PPI was constructed, and the hub genes including Insulin Like Growth Factor 1 (*IGF1*), C-X-C Motif Chemokine Ligand 10 (*CXCL10*), Interleukin 13 (*IL13*), Intercellular Adhesion Molecule 1 (*ICAM1*), G Protein Subunit Gamma Transducin 1 (*GNGT1*), Matrix Metallopeptidase 1 (*MMP1*), Neurexin 1 (*NRXN1*) and Matrix Metallopeptidase 3 (*MMP3*) were obtained. The expression levels *of CXCL10, IGF1, MMP3, MMP1, ICAM1,* and *IL-13* were significantly up-regulated in tumor tissues. High expression levels of *CXCL10, MMP3* and *ICAM1* predicted poor prognosis of NPC patients. *CXCL10* silencing suppressed NPC cell proliferation and migration.

Conclusions: *CXCL10* may serve as a potential key gene affecting NPC genesis and progression.

## INTRODUCTION

Nasopharyngeal carcinoma (NPC) originates in the upper and lateral nasopharyngeal cavity wall, which shows a high incidence in China and has the highest incidence among otorhinolaryngological cancers. NPC possesses a strong erosive activity and is prone to invasive expansion into surrounding organs and tissues, with frequent occurrence of regional lymph node metastasis (LNM). Due to the lack of specific early clinical manifestations, NPC is easily misdiagnosed as ear, nose and throat (ENT) diseases and is not paid attention to by patients. Therefore, most patients are diagnosed at stages III and IV. Because of the high recurrence rate and distant metastasis (DM) rate, locally advanced patients are mainly treated with radiotherapy supplemented with chemotherapy, biotherapy and other comprehensive treatment to improve the therapeutic effect.

Over the past few decades, RNA-sequencing (RNA-seq) has been applied in the diagnosis and molecular targeted therapy of numerous diseases. It is important to elucidate the functions of novel genes in diagnosing, treating and predicting the prognosis for NPC in clinic. A number of biomarkers in NPC have been reported in recent years. For instance, He et al. established a signature based on the long non-coding RNAs (lncRNAs) in serum to diagnose NPC. According to their results, *AL359062*, *MALAT1* and *AFAP1-AS1* were the candidate new serum biomarkers [1]. Luo et al. discovered that *FoxM1* was over-expressed in NPC tissues, and *FoxM1* induced the progression and cancer stem cell (CSC) features in NPC [2]. According to Fan et al., the *CORO1C* level was related to NPC metastasis; meanwhile, *CORO1C* overexpression enhanced the migration and invasion of NPC cells [3].

In the present study, NPC clinical samples and normal samples were collected for RNA-seq. In addition, we selected differentially expressed genes (DEGs), and conducted gene set enrichment analysis (GSEA). Through constructing the PPI network, the hub genes *CXCL10*, *GNGT1*, *IGF1*, *MMP3*, *MMP1*, *ICAM1*, *IL13*, and *NRXN1* were obtained. Later, *CXCL10* levels within NPC tissues were analyzed, and the function of *CXCL10* silencing in the proliferation, apoptosis, invasion and migration of cells was determined.

## MATERIALS AND METHODS

### Ethical statement

The Ethics Committee of The First Affiliated Hospital of Suzhou University approved our study protocol. Each participant provided the informed consent for participation.

### Patient samples and the clinicopathological features

We obtained samples from five NPC cases undergoing surgical treatment at the Department of Otolaryngology, The First Affiliated Hospital of Soochow University (Soochow, China) from May, 2016 to December, 2019 for RNA-seq. Additionally, six healthy controls were collected for RNA-seq, whereas additional 61 NPC patients and 61 healthy controls were collected for clinical verification. The clinicopathological characteristics of patients were recorded. The normal tissues were resected, and cancer tissues were carefully obtained from the middle of NPC. Each collected sample was frozen with liquid nitrogen and preserved under –80°C. At last, we acquired five NPC and six non-carcinoma samples for RNA-seq.

### RNA preparation

We utilized Trizol (Invitrogen, Carlsbad, CA, USA) for extracting the total RNA from normal and NPC samples, DEPC-treated water was utilized to re-dissolve total RNA, whereas NanoVue Plus spectrophotometry (GE Healthcare, Fairfield, CT, USA) was carried out to quantify total RNA. Agarose gel electrophoresis was conducted to evaluate the integrity of RNA, and gDNA Eraser (Takara, Tokyo, Japan) was also utilized for eliminating DNA contamination in accordance with specific protocols.

### RNA sequencing (RNA-Seq)

Trizol was used to grind the normal and NSP samples, whereas the RNeasy Kit (Qiagen) was employed to isolate total RNA in accordance with specific protocols. Besides, DNaseI was added to prevent tissues from mixing with genomic DNA. In addition, Agilent BioAnalyzer 2100 (Agilent, Santa Clara, CA, USA) was utilized to detect RNA purity. Moreover, we adopted the TruSeq RNA Access Library Prep Kit^®^ (Illumina, CA, USA) to prepare transcriptomic sequencing libraries. Further, we utilized the Illumina HiSeq 2000 instrument to perform 100 bp paired-end sequencing, as supported by Beijing Novogene Biological Information Technology Co., Ltd., (Beijing, China).

### Functional enrichment of DEGs

We utilized the clueGO plug-in of Cytoscape to carry out functional enrichment. ClueGO can be used for functional enrichment and classification of functional items with significant enrichment, including Gene Ontology (GO) terms and KEGG pathways [4]. During calculation, we determined the kappa-coefficient to reflect the functional associations of terms or pathways according to gene overlapping between different GO terms or pathways. In this study, the default value of kappa was 0.4. Entries with similar functions were presented by identical color. *P* < 0.05 was set as the significant enrichment threshold.

### PPI network construction

PPI can be obtained from the STRING database based on different data, including fusion, coexpression, text-mining and cooccurrence. This database comprehensively grades every PPI relation pair, with the score ranging from 0 to 1 [5]. A greater score indicates that the PPI relation is more creditable. Generally, 0.4 is used as the threshold of pooled score. In this study, we obtained PPI among DEGs from the STRING V10 database, established the PPI network and visualized it using Cytoscape.

### Immunohistochemistry (IHC)

After deparaffinization, fixed sections were rehydrated with gradient xylene and ethanol, and incubated with 0.3% H_2_O_2_ solution under ambient temperature for 30 min. Sodium citrate solution (pH 6.0) was used to retrieve the antigen. Thereafter, each section was blocked using the BSA solution for reducing the nonspecific binding. Later, each section was subjected to FGF5 antibody staining, and incubation using secondary antibody (Envision, Gene Technology, Shanghai, China) for visualization. Later, DAB was employed for color developing of each slide, whereas hematoxylin was used for counterstaining.

### Cell culture and transfection

The CNE-2 and 5–8F NPC cells were provided by Central Laboratory of Soochow University (Suzhou, China) and cultivated within the RPMI-1640 medium (Gibco, Gaithersburg, MD, USA, A1049101) that contained 10% fetal bovine serum (FBS; Gibco, 10099141) under 37°C and 5% CO_2_ conditions.

### Cell lines and cell culture

CNE-2 and 5–8F cells were obtained from the cell bank at the Chinese Academy of Sciences (Shanghai, China). Afterwards, all cells were cultivated within the RPMI-1640 medium that contained 10% FBS (Gibco, 10099141) under the humidified, 37°C and 5% CO_2_ conditions.

### Cell transfection

The siCXCL10-1 (5′-ACTGCCATTCTGATTTGCTGCCTTA-3′), siCXCL10-1 (5′-GCTGCCTTATCTTTCTGACTCTAAG-3′) and negative control (siNC, 5′-CGGTATGCCATGCCTACGCTATCGAAC-3′) were provided by GenePharma (Shanghai, China). After culture within the antibiotics-free complete medium for 24 h pre-transfection, cells were rinsed by PBS, followed by temporary transfection using siNC or siFGF5 (50 nmol/L) using Lipofectamine 2000 (Invitrogen, USA) in accordance with specific protocols. At 24 h post-transfection, we conducted qRT-PCR for validating the *CXCL10* level.

### RNA Isolation and qRT-PCR

We utilized Trizol reagent (Takara, Japan) for extracting total tissue RNA from primary tumor and non-carcinoma samples, respectively. Later, the extracted total RNA was used to prepare cDNA by reverse transcription using the PrimeScript RT-PCR kit (Takara, Japan) in accordance with specific protocols. RT-PCR was carried out by the ABI7500 (Applied Biosystem) thermal cycler with the standard SYBR Green PCR kit (Takara). In this study, the primers used were shown below: for *CXCL10*, 5′-AAGCAGTTAGCAAGGAAAGG-3′ and 5′-GTAGGGAAGTGATGGGAGAG-3′; for *GNGT1*, 5′-GAAATTGGTTATCGTGGGAT-3′ and 5′-TCACTTCTTTCTTGAGCTGGT-3′; for *IGF1*, 5′-AGGAGGCTGGAGATGTATTG-3′ and 5′-GTGTTCTTGTTGGTAGATGGG-3′; for *MMP3*, 5′-GGTCTCTTTCACTCAGCCA-3′ and 5′-GGGTCTCAGGGGAGTCA-3′; for *MMP1*, 5′-GCTACACGGATACCCCAA-3′ and 5′-CTCAGAAAGAGCAGCATCG-3′; for *ICAM1*, 5′-AAACACTAGGCCACGCATC-3′ and 5′-CCCACCACTTCCCCTCT-3′; for *IL13*, 5′-CAGCTCAGGCACACTTCTT-3′ and 5′-CTAGCAGCCACAGTCTTCC-3′; for *NRXN1*, 5′TCCTCGGGTTAAGAAATGG-3′ and 5′-CCTTGGATGCTTGTGAATG-3′; for *CXCR3*, 5′-ACAAGCACCAAAGCAGAGG-3′ and 5′-CTGGGCAGCAGCACTTAC-3′; and for *GAPDH*, 5′-CACCCACTCCTCCACCTTTG-3′ and 5′-CCACCACCCTGTTGCTGTAG-3′. The 2^–ΔΔCt^ approach was adopted for calculating relative gene level, with GAPDH as the endogenous control.

### Cell proliferation assay

In the present work, we applied MTT assay for assessing cell proliferation according to specific instructions (Invitrogen, M6494). Briefly, corresponding vectors were transfected into CNE-2 and 5–8F cells, and incubated for 0, 24, 48 and 72 h, separately. After 20 μL MTT solution was added to incubate cells for 4 h, we discarded supernatants. Afterwards, the products were dissolved through the addition of dimethyl sulfoxide (100 μL, DMSO). At last, absorbance (OD) value was detected by using the microplate reader (Bio-Rad, Richmond, CA, USA, 168-1000XC) at 490 nm.

### Cell apoptosis assay

The propidium iodide (PI)/Annexin-V Cell Apoptosis Kit (Invitrogen, V13245) was utilized to analyze C666-1 and 5–8F apoptosis rates according to specific protocols. Briefly, vectors were transfected into C666-1 and 5–8F cells and later cultured for 48 h. Then, cells were stained with FITC-Annexin V and PI. At last, we conducted flow cytometric analysis (FACSCantoII, 338960; BD Biosciences, San Jose, CA, USA) for determining cell apoptosis rate.

### Transwell invasion assay

The Matrigel-coated (BD, Franklin Lakes, NJ, USA) Transwell chambers (Costar, Manassas, VA, USA) that contained 8-um polycarbonate filters were used for the invasion assay. At 48 h post-transfection, cells cultured within the FBS-free RPMI-1640 (200 μl) were added into the Matrigel (1 mg/ml, 30 μl)-coated upper chamber. Meanwhile, FBS-containing RPMI-1640 was added into the lower chamber, which served as the chemo-attractant. After incubation for 24 h, we scraped cells on membrane surface, then those scraped cells were subjected to PBS rinsing, 100% methanol fixation, and Giemsa dye staining.

### Scratched wound healing assay

Cells (1 × 10^6^ cells/well) were inoculated within the 6-well plates in the wound healing assay. When cells reached about 90% confluence, we used the pipette tip (1 ml) to made a wound on the surface of the cell monolayer. Microscopy was conducted to obtain images at 0 and 48 h, respectively, for assessing the wound closure rate. The ImageJ software (NIH, Bethesda, MD, USA) was applied in measuring inter-edge distance. To be specific, the wound closure rate was determined by the following formula: (scratch width at 0 h- scratch width at 24 h/48 h)/scratch width at 0 h × 100%.

### Statistical analysis

We used the Fisher’s exact test to assess the clinicopathological features, such as sex, age, tumor stage, tumor size, and differentiation. Animal and functional experiments *in vitro* were carried out to assess the expression level by student’s *t*-test. SPSS18.0 (SPSS, Inc., Chicago, IL, USA) was utilized in all statistical analyses (two-sided). *P* < 0.05 was set as the significance level.

## RESULTS

### Raw data preprocessing and DEGs screening

**Figure 1:**
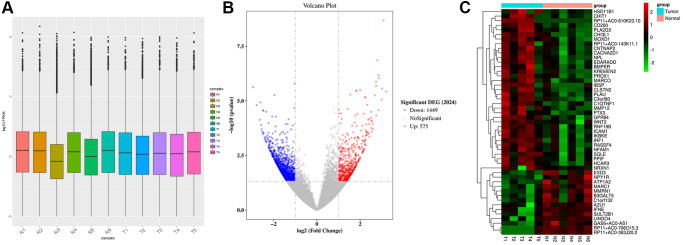
**Raw data preprocessing and DEGs screening.** (**A**) The distribution of gene expression levels in the samples was relatively uniform. (**B**) A total of 2024 DEGs were obtained between Normal group (N, *n* = 6) and Tumor group (T, *n* = 5). 1449 DEGs were down-regulated, and 575 DEGs were up-regulated. (**C**) Heatmap shows the DEGs.

Gene expression was uniformly distributed within samples after preprocessing and the processed data were utilized in later analyses (Figure 1A). We classified 11 samples into 2 groups, including Tumor group (T, *n* = 5) and Normal group (N, *n* = 6). ANOVA was performed for analyzing DEGs in both groups, yielding altogether 2024 DEGs (Figure 1B). Among the DEGs, 1449 were down-regulated while 575 were up-regulated. The cluster analysis of DEGs can be observed from Figure 1C. The 10 respective most significantly up-regulated and down-regulated DEGs are presented in Table 1. Of them, the top 10 up-regulated DEGs were *RP11-810K23.10*, *CHI3L1*, *KREMEN2*, *NRXN1*, *CHIT1*, *IBSP*, *CLSTN2*, *MMP12*, *WNT2*, and *CNTNAP2*. The 10 most significantly down-regulated DEGs included *RP11-706O15.3*, *AZU1*, *RP11-363J20.2*, *B3GALT5*, *ABO*, *GJB6*, *RP11-501J20.5*, *ATP1A2*, *RP11-646E20.6*, and *CYP2G1P*.

**Table 1:** The respective most significantly up-regulated and down-regulated DEGs.

**Genes**	**Fold change**	***P* value**
Upregulated		
RP11-810K23.10	3.21	3.76E-06
CHI3L1	3.08	2.08E-09
KREMEN2	3.02	2.72E-06
NRXN1	2.99	8.70E-06
CHIT1	2.86	8.97E-07
IBSP	2.85	2.65E-05
CLSTN2	2.84	6.63E-07
MMP12	2.80	1.39E-07
WNT2	2.75	4.73E-05
CNTNAP2	2.74	2.07E-06
Downregulated		
RP11-706O15.3	–2.94	2.36E-06
AZU1	–2.78	1.57E-05
RP11-363J20.2	–2.63	7.32E-05
B3GALT5	–2.61	1.17E-05
ABO	–2.58	1.27E-04
GJB6	–2.55	1.97E-04
RP11-501J20.5	–2.53	2.56E-04
ATP1A2	–2.50	2.74E-05
RP11-646E20.6	–2.50	3.35E-04
CYP2G1P	–2.47	2.80E-04

### Functional enrichment of NPC related genes

**Figure 2:**
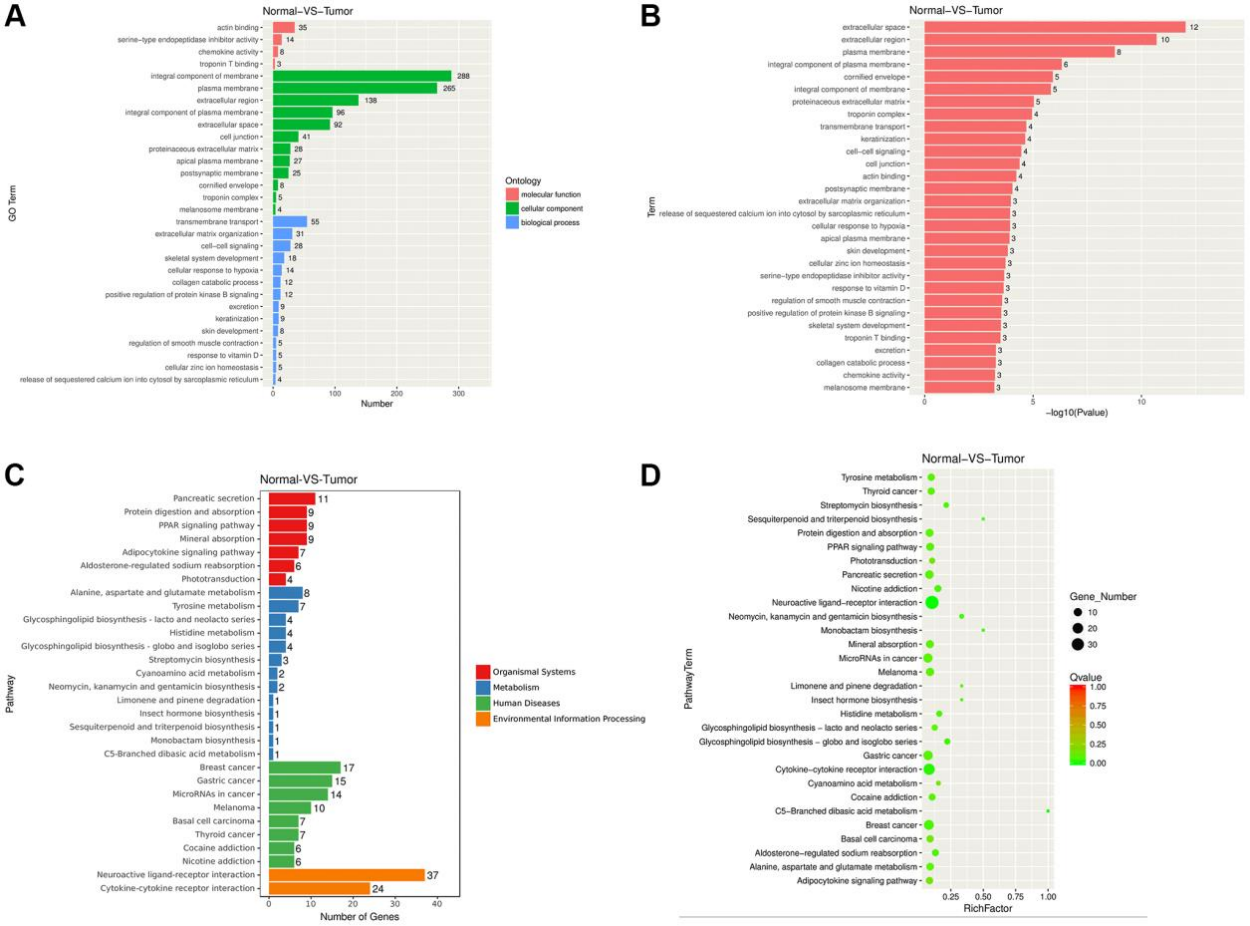
**Functional enrichment of NPC related DEGs.** (**A**–**B**) DEGs were enriched in GO terms. (**C**–**D**) DEGs were enriched in KEGG pathways.

This study also examined the roles of DEGs through GO and KEGG analyses, respectively. Typically, GO enrichment of DEGs directly reflects the enrichment of GO terms including biological process (BP), cell component (CC) and molecular function (MF). We selected 30 most significant GO terms, as shown in Figure 2A. According to Figure 2A, DEGs were enriched in extracellular space (CC), transmembrane transport (BP), and actin binding (MF). We obtained 30 significantly enriched GO terms, including plasma membrane, extracellular region, extracellular space, cornified envelope, integral component of plasma membrane. As observed from the figure of *P*-value, DEGs were enriched in pathways such as extracellular space, extracellular region, and plasma membrane pathway (Figure 2B). We then performed KEGG pathway enrichment analysis, which revealed that DEGs were related to pathways such as Pancreatic secretion (organismal systems), Alanine, aspartate and glutamate metabolism (metabolism), Breast cancer (human diseases), and Neuroactive ligand-receptor interaction signaling pathways (environmental information processing) (Figure 2C). Meanwhile, as suggested from the KEGG pathway scatter plot, DEGs were mostly related to Neuroactive ligand-receptor interaction, Cytokine receptor interaction, and protein digestion and absorption (Figure 2D).

### PPI network of NPC related genes

**Figure 3:**
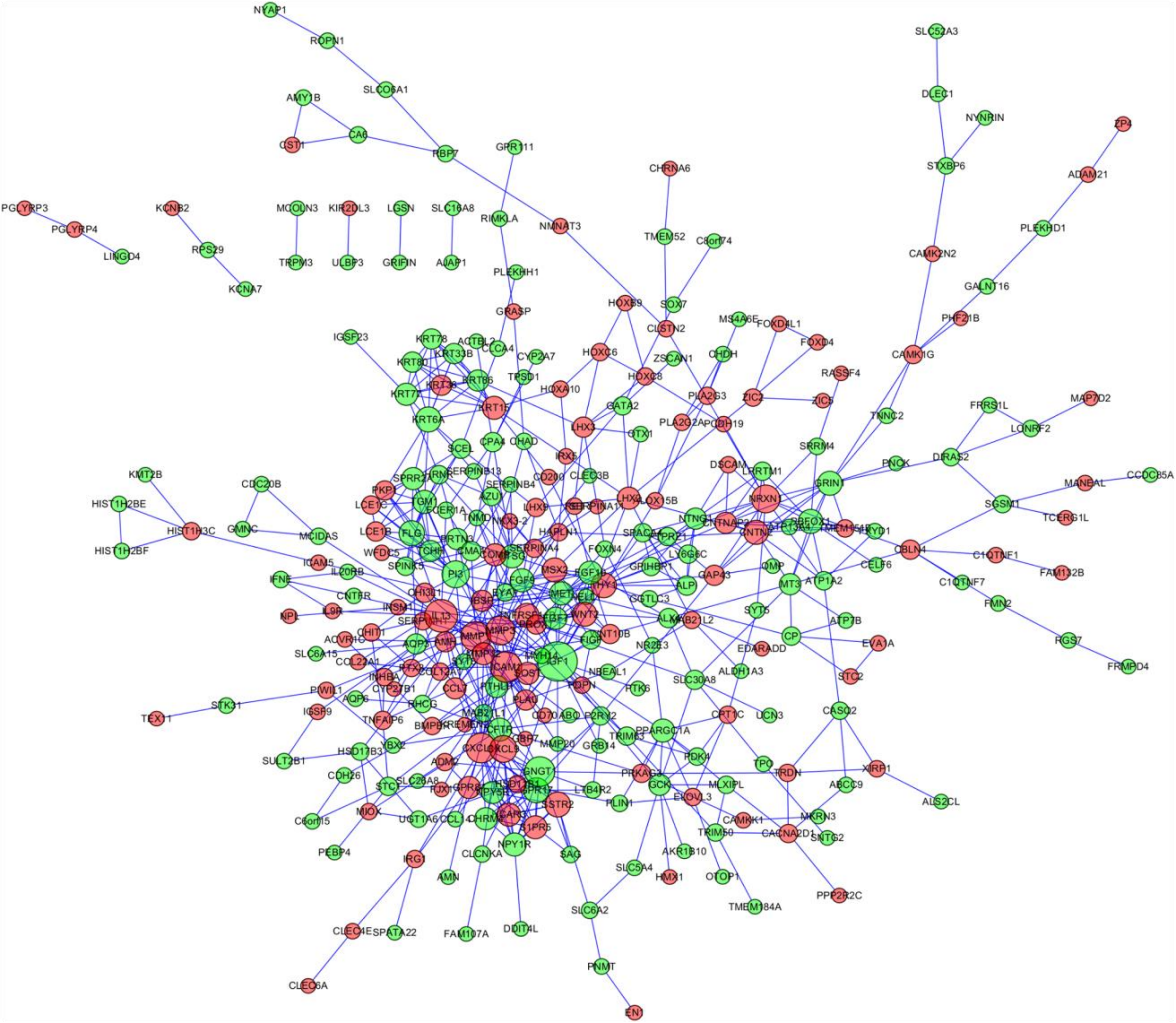
Protein-protein international (PPI) network of nasopharyngeal carcinoma related genes.

Later, the NPC-related DEGs were extracted from the STRING V10 database to construct a PPI network. Figure 3 presents the established PPI. Typically, the 8 most significant nodes within the PPI network were Insulin Like Growth Factor 1 (IGF1, degree = 27), Interleukin 13 (IL13, degree = 20), Intercellular Adhesion Molecule 1 (ICAM1, degree = 18), G Protein Subunit Gamma Transducin 1 (GNGT1, degree = 17), C-X-C Motif Chemokine Ligand 10 (CXCL10, degree = 17), Matrix Metallopeptidase 1 (MMP1, degree = 17), Matrix Metallopeptidase 3 (MMP3, degree = 16), and Neurexin 1 (NRXN1, degree = 15). These hub nodes had the highest degree and edge values, indicating their irreplaceable roles in NPC. Therefore, further experiments should be performed to investigate the above genes for their clinical meaning.

### Expression levels of the top 8 DEGs in NPC patients

**Figure 4:**
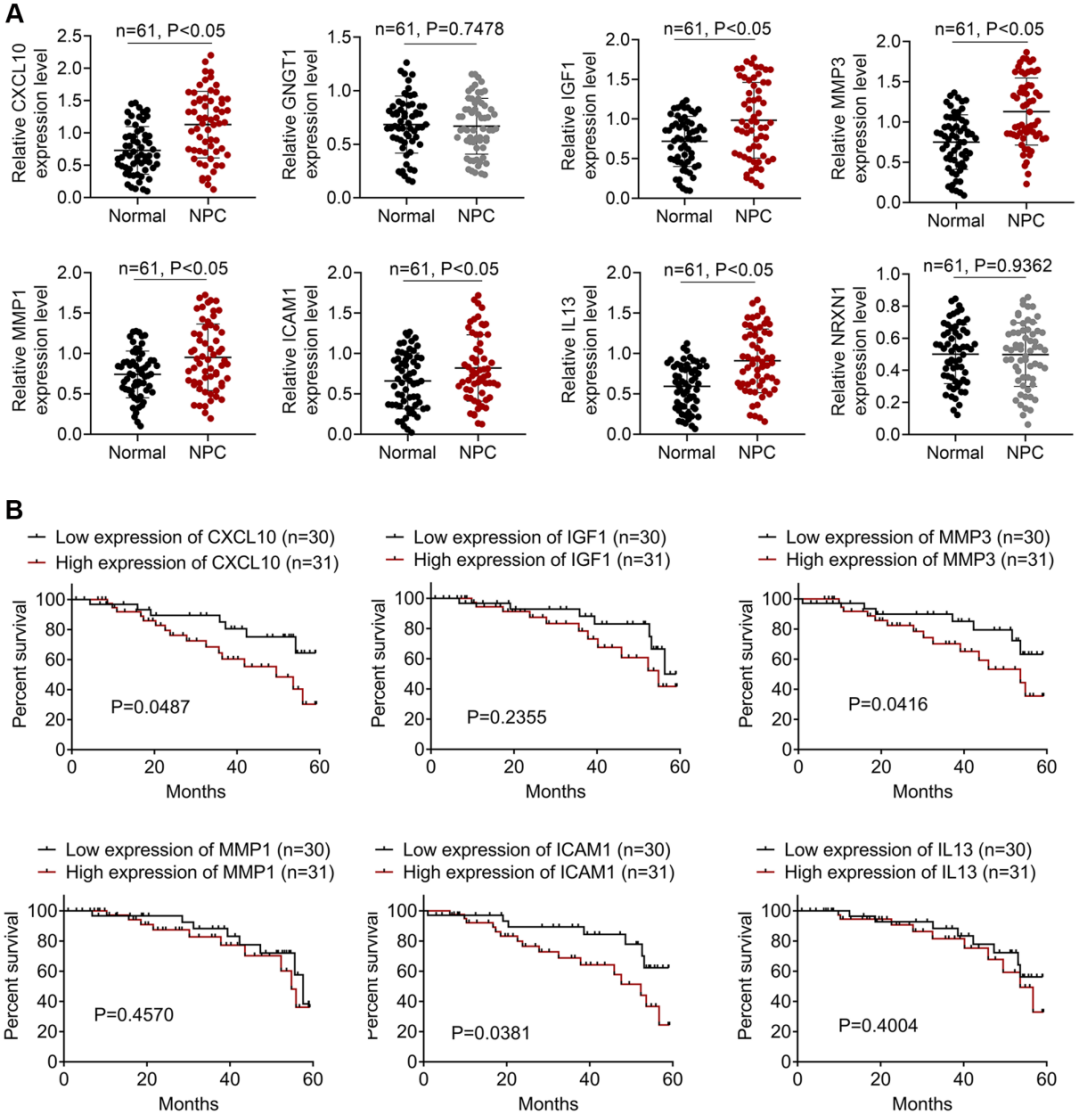
**The expressions of DEGs in NPC patients.** (**A**) The qRT-PCR assay was employed to detect the expressions of *CXCL10*, *GNGT1*, *IGF1*, *MMP3*, *MMP1*, *ICAM1*, *IL13*, and *NRXN1*. (**B**) The survival analysis of I*GF1, IL13, ICAM1, CXCL10, GNGT1, MMP1, MMP3*, and *NRXN1* in NPC patients.

We collected 61 pairs of NPC and normal samples to verify the expression levels of the top 8 DEGs. Levels of *CXCL10, GNGT1, IGF1, MMP3, MMP1, ICAM1, IL13*, and *NRXN1* were measured through qRT-PCR assay, as presented in Figure 4A. As a result, *CXCL10, IGF1, MMP3, MMP1, ICAM1,* and *IL-13* levels were markedly up-regulated within NPC samples relative to the non-carcinoma samples (*P* < 0.05). While, difference in the expression of GNGT1 and NRXN1 was not significant in NPC samples compared with non-carcinoma samples. Additionally, the survival analysis of *CXCL10*, *IGF1*, *MMP3*, *MMP1*, *ICAM1*, and *IL-13* was performed. The results showed that high expression levels of *CXCL10*, *MMP3* and *ICAM1* predicted poor prognosis of NPC patients (Figure 4B). The clinical significance of *MMP-3* and *ICAM1* in NPC is already reported [6], so this study focused on *CXCL10*.

### CXCL10 knockdown suppressed NPC cell proliferation and invasion

**Figure 5:**
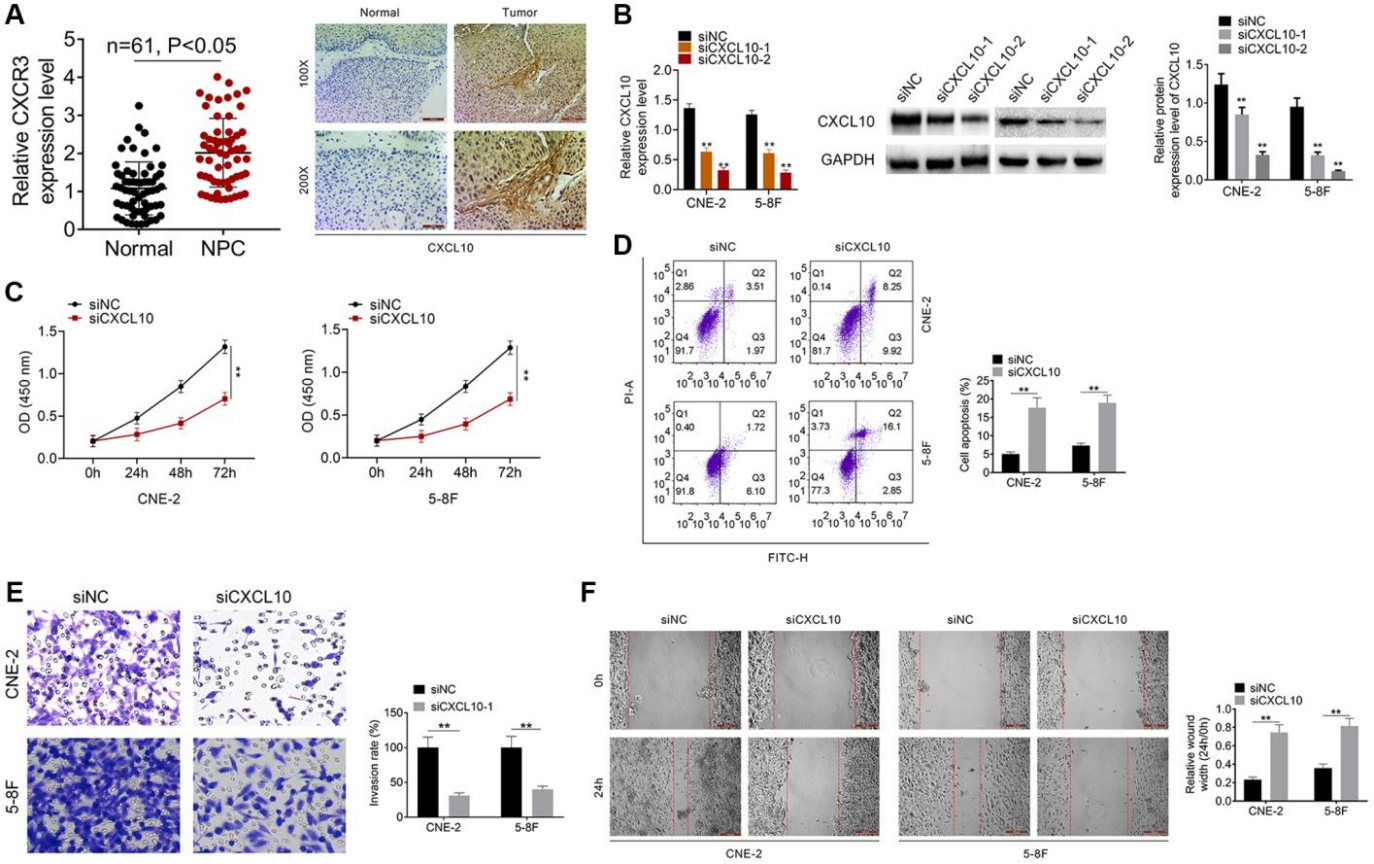
**Knockdown of CXCL10 inhibited cell proliferation and invasion of NPC cells.** (**A**) The expression of CXCR3 was examined by qRT-PCR assay. The expression of CXCL10 in NPC tumor tissue and normal tissue was examined by IHC analysis. (**B**) CNE-2 and 5–8F cells were transfected with siNC, siCXCL10-1 or siCXCL10-2, and RT-PCR was performed to examine the CXCL10 expression. (**C**) Cell proliferation of CNE-2 and 5–8F cells was identified by CCK8 assay. (**D**) Cell apoptosis of CNE-2 and 5–8F cells was evaluated by FCM. (**E**) Cell invasion was tested by wound healing assay. (**F**) Cell migration was detected by Transwell assay. ^**^P < 0.01 vs siNC group.

This study detected the CXCR3 (CXCL10 receptor) expression, and the result showed that the CXCR3 was over-expressed in NPC samples (Figure 5A). IHC assay was conducted for identifying *CXCL10* levels within NPC samples and non-carcinoma samples. As a result, *CXCL10* over-expression was detected within NPC samples (Figure 5A). As presented in Table 2, the high *CXCL10* expression showed positive correlation with TNM stage, T stage and N stage. After siNC, siCXCL10-1 or siCXCL10-2 was transfected into 5–8F and CNE-2 cells, RT-PCR was performed to examine the transfection efficiency (Figure 5B). At last, we selected siCXCL10-2 for further experiments. Cell proliferation, apoptosis, invasion and migration were assessed. According to our results, down-regulation of *CXCL10* remarkably suppressed NPC cell proliferation, migration and invasion, and promoted their apoptosis relative to cells transfected with siNC (*P* < 0.01, Figure 5C–5F).

**Table 2:** The relationship between CXCL10 expression and clinicopathologic characteristics of NPC patients.

**Characteristics**	**Number of patients**	**CXCL10**	**CXCL10**	***P* value**
		**Low expression** **(<median)**	**High expression** **(≥median)**	
Number	61	29	32	
Ages				0.452
<50	31	14	17	
≥50	30	15	15	
Gender				0.357
Female	29	15	14	
Male	32	14	18	
TNM stage				0.048
I-III	30	18	12	
IV	31	11	20	
T stage				0.015
T1-T2	28	18	10	
T3-T4	33	11	22	
N stage				0.045
N0-N1	32	19	13	
N2-N3	29	10	19	

## DISCUSSION

In this work, we performed RNA-seq and bioinformatic analysis to screen the crucial biomarkers affecting the occurrence and development of NPC. The up-regulated and down-regulated DEGs were obtained at first, and then GSEA analysis was processed. Several pathways including extracellular space [7], extracellular region [8], plasma membrane [9], and integral component of plasma membrane [10] were closely associated with tumor genesis, growth and metastasis. Loom et al. investigated that extracellular space was an important compartment for malignant energetic catalysis and therapeutic targeting [7]. It is reported that extracellular region plays a crucial role in oncogenic events of tropomyosin receptor kinase (TRK) in several cancers [8]. In addition, plasma membrane and integral component of plasma membrane pathways are also proved to regulate cancer progression on breast cancer [11]. Some cancer-related KEGG pathways are identified, such as breast cancer, Cytokine receptor interaction [12], and protein digestion and absorption [13]. A lot of studies report that DEGs including mRNAs and non-coding RNAs in many kinds of cancers are enriched in KEGG Cytokine receptor interaction pathway [14, 15]. And, Han et al. identified abnormally methylated DEGs and pathways associated with NPC, and they found that DEGs were also enriched in protein digestion and absorption pathway [16]. Furthermore, we constructed the PPI network, and identified *IGF1*, *IL13*, *ICAM1*, *CXCL10*, *GNGT1*, *MMP1*, *MMP3*, and *NRXN1* as the hub genes. Our clinical data verified that the expression levels of *IGF1*, *IL13*, *ICAM1*, *CXCL10*, *MMP1*, and *MMP3* were up-regulated in NPC tissues. *IGF1* is a polypeptide that is structurally similar to human pro-insulin, which is one of the pathogenetic factors resulting in obesity and other diseases [17]. *IGF1* is also a high risk factor for several cancers, such as prostate cancer [18], breast cancer [19], lung cancer [20], gastric cancer [21], and colon cancer [22]. The high serum IGFBP-1-to-IGF-1 ratio is related to the adverse prognostic outcome for NPC [23]. Wang et al. reported that the loss of miR-206 reduced the radiosensitivity of NPC by targeting *IGF-1* [24]. *IL13* serves as an immunoregulatory cytokine, which is closely related to cancer genesis by impacting the tumor immune surveillance [25]. *ICAM1*, *MMP1*, and *MMP3* are the well-known biomarkers participating in regulating cancer cell migration and invasion. *ICAM1* is involved in tumor cell adhesion to vascular endothelium or neutrophils, and mediates the hematogenous and lymph node metastasis of malignant tumors [26, 27]. The high expression levels of *MMP1* and *MMP3* enhance cancer cell migration and invasion, and predict poor prognosis for NPC [28, 29]. *CXCL10* is over-expressed in several kinds of cancers, and it is reported to regulate cancer progression, such as colorectal cancer (CRC) [30], cervical cancer [31], ovarian cancer (OC) [32], and breast cancer (BC) [33, 34]. However, the function of *CXCL10* in NPC is still unknown. As revealed by our results, *CXCL10* was over-expressed in NPC tissues, and the up-regulated *CXCL10* expression showed positive correlation with TNM stage, T stage and N stage. The above findings displayed that *CXCL10* possibly had oncogenic effect in NPC. CXCR3, as a CXCL10 receptor, was also up-regulated in NPC samples. Thereafter, we performed cellular functional assays to identify the role of *CXCL10* in tumor cell activities. As a result, the down-regulated *CXCL10* expression suppressed CNE-2 and 5–8F proliferation, migration and invasion, but enhanced their apoptosis. These data implied that CXCL10 might serve as a candidate biomarker for molecular diagnosis and targeted therapy of NPC.

Certain limitations should be noted in the present work. First of all, this study did not fit the overall survival time or the gene expression levels. Secondly, the present work involved few clinical indicators and sample size. At last, the detailed molecular mechanism by which CXCL10 affected NPC cell proliferation and invasion should be further studied. Our next study will emphasize the above issues. To sum up, CXCL10 is a candidate key gene affecting NPC genesis and progression. This work sheds new lights on the molecular diagnosis and targeted therapy of NPC.
